# SUPPORTING FAMILY CAREGIVERS THROUGH PATIENT TRANSITIONS FROM HOSPITAL TO HOME: THE CARE ACT AND BEYOND

**DOI:** 10.1093/geroni/igz038.2280

**Published:** 2019-11-08

**Authors:** Joan M Griffin, Diane Holland, Lauren R Bangerter, Cory Ingram, Ellen Wild, Erin Taylor, Rachel Gentes, Catherine Vanderboom

**Affiliations:** 1 Mayo Clinic, Rochester, United States; 2 Mayo Clinic, Rochester, Minnesota, United States

## Abstract

To date, nearly 40 U.S. states have passed the Caregiver Advise Record and Enable (CARE) Act to support family caregivers during transitions from hospital to home. These laws require hospitals to: 1) record designated family caregivers’ name in a patient medical records during a hospital stay; 2) inform the caregiver when the patient will be discharged; 3) provide education and instructions on care tasks needed for post-hospital discharge. Transitions from hospital to home are often fraught with adverse event risk and poor continuity of care, especially for rural patients with progressive life-limiting conditions. The CARE ACT has potential to address healthcare system factors that are sometimes attributable to poor transitions in care. Our presentation focuses on preliminary findings from an intervention that provides teaching, guidance, and counseling to caregivers caring for an individual receiving palliative care in the hospital and transitioning home to a rural setting. Findings suggest that the CARE ACT may be vital, but not fully sufficient, for successful transitions. Additional targets for caregiver interventions to improve transitions for this subset of very ill patients include: 1) attend to caregivers’ medical and non-medical needs which may impact their capacity to provide care; 2) advise clinical teams to communicate truthfully about prognosis and likely outcomes when creating post-hospital plans of care; 3) encourage caregivers to identify and engage services early in the transition; 4) urge caregivers to draw on multiple sources for social support. Findings can help optimize the CARE Act implementation, particularly in states with large rural populations.

